# Rapid Stereomicroscopic Imaging of HER2 Overexpression in *Ex Vivo* Breast Tissue Using Topically Applied Silica-Based Gold Nanoshells

**DOI:** 10.1155/2012/291898

**Published:** 2012-10-22

**Authors:** Lissett R. Bickford, Robert J. Langsner, Joseph Chang, Laura C. Kennedy, Germaine D. Agollah, Rebekah Drezek

**Affiliations:** ^1^Department of Bioengineering, Rice University, 6100 Main Street, MS 142, Houston, TX 77005, USA; ^2^School of Biomedical Engineering and Sciences, Virginia Polytechnic Institute and State University, Blacksburg, VA 24061, USA; ^3^Department of Mechanical Engineering, Virginia Polytechnic Institute and State University, Blacksburg, VA 24061, USA; ^4^School of Medicine, University of California San Francisco, San Francisco, CA 94143, USA; ^5^School of Medicine, Baylor College of Medicine, Houston, TX 77030, USA; ^6^Department of Chemistry and Process Development, Nanospectra Biosciences, Inc., Houston, TX 77054, USA; ^7^Department of Electrical and Computer Engineering, Rice University, Houston, TX 77005, USA

## Abstract

Tumor margin detection for patients undergoing breast conservation surgery primarily occurs postoperatively. Previously, we demonstrated that gold nanoshells rapidly enhance contrast of HER2 overexpression in *ex vivo* tissue sections. Our ultimate objective, however, is to discern HER2 overexpressing tissue from normal tissue in whole, nonsectioned, specimens to facilitate rapid diagnoses. Here, we use targeted nanoshells to quickly and effectively visualize HER2 receptor expression in intact *ex vivo* human breast tissue specimens. Punch biopsies of human breast tissue were analyzed after a brief 5-minute incubation with and without HER2-targeted silica-gold nanoshells using two-photon microscopy and stereomicroscopy. Labeling was subsequently verified using reflectance confocal microscopy, darkfield hyperspectral imaging, and immunohistochemistry to confirm levels of HER2 expression. Our results suggest that anti-HER2 nanoshells used in tandem with a near-infrared reflectance confocal microscope and a standard stereomicroscope may potentially be used to discern HER2-overexpressing cancerous tissue from normal tissue in near real time and offer a rapid supplement to current diagnostic techniques.

## 1. Introduction

Currently, breast cancer is the second leading cause of cancer-related deaths in women, and it accounts for approximately one-third of all cancers diagnosed in women in the United States [1]. To reduce cancer recurrence and progression, cancerous tissue must be completely eliminated, regardless of grade [2]. Surgical breast cancer therapy focuses on removing the primary tumor and identifying the possibility of metastatic disease from the evaluation of sentinel lymph nodes. Although some patients may require modified radical mastectomy, many patients with less-advanced breast cancer elect breast-conserving surgery. The presence of a positive surgical margin during these surgeries has been associated with lower rates of patient survival [3]. Due to residual cancer cells being left in many patients that undergo breast conservation therapy, as many as 40% of patients have experienced local breast cancer recurrence near the site of the original tumor [4]. Intraoperative treatment decisions are, therefore, absolutely critical.

Presently, intraoperative tumor margin detection occurs primarily in specialized tertiary centers, such as The University of Texas M.D. Anderson Cancer Center (MDACC). In these centers, the resected tissue receives a preliminary evaluation by a pathologist while the patient remains in the operating room; if necessary, additional tissue can be removed until the pathologist determines that the tumor margins are negative. In community hospitals, however, pathologic analysis of excised tissue only occurs postoperatively [5]. Patients who consequently have positive tumor margins must return for surgical reexcision and receive increased doses of adjuvant radiation therapy [6, 7]. Thus, the existence of positive tumor margins portends additional risks and costs to the patient. Due to the existing limitations of current intraoperative tumor margin detection, there is an opportunity to develop superior diagnostic tools to assist in reducing the recurrence and progression of cancer due to inadequate tissue removal during primary surgery. 

 While histologic analysis remains the gold standard for tumor margin assessment, the macroscopic evaluation of whole, nonsectioned tissue specimens may also be used to provide an intraoperative estimate of tumor margin status prior to subsequent processing. This would be an invaluable tool in hospitals without onsite pathology suites. Macroscopic visualization of questionable tissue is attractive for enhancing the sensitivity and specificity of tumor margin delineation: if the number of suspicious regions that require further microscopic processing can be reduced, surgeons and pathologists can focus their attention and resources on areas that remain inconclusive. Currently, macroscopic evaluation only occurs for breast cancer specimens that involve microcalcifications or nonpalpable masses and does not occur for palpable breast masses [8]. For nonpalpable masses that have been resected, radiographic images are used to determine the extent of the breast disease and the proximity to the resected margins. Although specimen radiography appears to increase the accuracy of tumor margin detection, limitations have been noted. For instance, microcalcifications that appear as tumor on radiographic images may actually be areas of lymphocytic accumulation [9]. The use of contrast agents targeted to specific biomarkers associated with disease may present an opportunity to increase the sensitivity and specificity of macroscopic evaluations.

 In preceding studies, we confirmed that silica-based gold nanoshells targeted to the Human Epidermal growth factor Receptor 2 (HER2) could be used for the rapid contrast enhancement of both cells [10] and tissue sections [11] which overexpress HER2 biomarkers. While gold nanoshells can be conjugated to a variety of biomarkers [12, 13], we have selected HER2 due to its association with increased cancer aggression, recurrence, and progression when amplified [14, 15]. Amplification of this cell-surface bound tyrosine kinase receptor occurs in up to a quarter of all human breast cancer cases [16]. Importantly, using biomarkers for tumor margin detection has recently been shown to better identify patients at high risk of cancer recurrence over standard histological analysis [17].

 To facilitate prompt tumor margin detection intraoperatively, the ability to assess tumor margins without physical sectioning is highly desirable as sectioning may incur significant time to the surgical procedure [5]. Thus, in this study, we advance our previous findings by examining the ability to rapidly target HER2 receptors in intact *ex vivo* human breast tissue specimens without sectioning. We first confirm the predominance of the surface targeting needed to identify the tumor margins and preferential labeling of HER2-positive tissue using two photon and hyperspectral imaging. Then, we demonstrate that anti-HER2-targeted gold nanoshells can be used as rapid diagnostic imaging agents for HER2 overexpression in intact breast tissue specimens using a standard stereomicroscope and confirm these results through reflectance confocal microscopy and immunohistochemistry.

## 2. Materials and Methods

### 2.1. Nanoshell Fabrication and Antibody Conjugation

**Figure 1:**
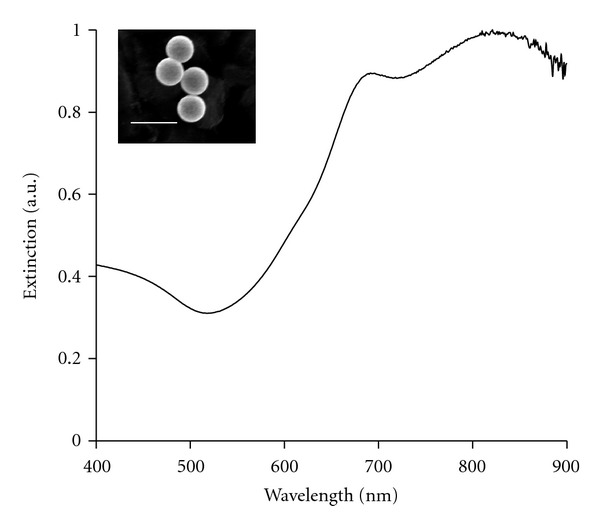
Measured extinction spectra of nanoshells with an average core diameter of 276 nm and average shell thickness of 19 nm. Insert depicts corresponding image from scanning electron microscopy. Scale bar represents 500 nm.

Nanoshells were fabricated as formerly described [18–20], and only a brief summary will be provided here. Silica cores were made using the Stöber method [21], in which tetraethyl orthosilicate was reduced in the presence of ammonium hydroxide dissolved in 200 proof ethanol. The surfaces of the cores were then modified by reaction with aminopropyltriethoxysilane (APTES) to functionalize reactive amine groups on the surface. The final particles were measured by dynamic light scattering (DLS) to have an average diameter of 276 nm. Next, gold colloid (*∼*1–3 nm diameter) was fabricated and adsorbed onto the surface of the silica cores via the amine groups to form gold nucleation sites [22]. To fully cover the surface of the silica cores, additional gold was added to these nucleation sites via a reduction reaction in which hydrogen tetrachloroaurate trihydrate (HAuCl_4_3H_2_O) was dissolved in potassium carbonate and then added with formaldehyde to help reduce the gold. After the gold layer over the silica cores was formed, the spectrum of the final nanoshell solution was visualized using a UV-VIS spectrophotometer (Varian Cary 300) (Figure 1).

To determine the concentration of nanoshells in solution, the absorption, scattering, and extinction coefficients were determined using Mie theory. The average nanoshell diameter, as validated by scanning electron microscopy (SEM), was 314 nm with a peak surface plasmon resonance at 840 nm. The concentration of the working nanoshell solution was approximately 2.0 × 10^9^ particles/mL. 

Nanoshells were targeted to biological HER2 antigens by linking the surfaces of the nanoshells to anti-HER2 antibodies using previously described methods [18]. Prior to beginning experimental studies, nanoshells were incubated with an anti-HER2-linker cocktail [18] for 2 hours at 4°C. To ensure nanoparticle stabilization in biological media, the nanoshells were next incubated with a 1 mM polyethylene glycolthiol solution (PEG-SH, MW = 5 kD, Nektar) for 12–16 hours at 4°C. Next, unbound antibodies and excess PEG-SH were removed from the nanoshells by centrifugation. Prior to experimental studies, the nanoshells were resuspended in antibody diluent (IHC World, pH 7.4) by gentle pipetting to a final volume of 165 *μ*L. 

### 2.2. *Ex Vivo* Human Breast Tissue Specimens

Normal and cancerous (HER2-negative and HER2-positive) breast tissue specimens were supplied by the Cooperative Human Tissue Network (CHTN) through a protocol approved by the Institutional Review Board (IRB). Tissues were designated as normal or cancerous by pathologists at the medical centers where the tissue samples were obtained. Additionally, HER2 status was previously determined by pathologists at the respective medical centers prior to the patients undergoing any form of medical treatment.

Before use, samples were thawed briefly in a 37°C water bath and cut on a disposable cutting board using a 5 mm punch biopsy to maintain size consistency. At least two punch biopsies were taken from each specimen for control and experimental conditions. Each cut specimen used was 5 mm in diameter with an average thickness of 1 mm. Tissue samples were subsequently incubated in prewarmed antibody diluent for 1 minute at room temperature with gentle agitation in a 24-well plate. After prerinsing, the samples were incubated in either antibody diluent or the aforementioned targeted-nanoshell cocktail in polyethylene sample vials (Sigma Aldrich). The vials were placed on a nutator in an incubator at 37°C for 5 minutes. After incubation, the tissue samples were removed from the vials and rinsed 3 times in 1x PBS briefly in a 24-well plate. Samples were moved to a clean well of 1x PBS prior to imaging. 

### 2.3. Two-Photon Imaging of Human Breast Tissue Specimens

Both HER2-positive and HER2-negative cancerous samples were evaluated for surface labeling of HER2-targeted nanoshells by employing two-photon imaging of intact breast tissue specimens. Samples were placed directly on a glass coverslip (Fisher Scientific), and an additional coverslip was placed on top of the tissue in order to facilitate moderate tissue compression. For image acquisition, a Zeiss multi-photon confocal microscope (LSM 510 META NLO) was used in tandem with a Coherent Chameleon femtosecond-pulsed, mode-locked Ti: sapphire laser. This system was set to operate as formerly described [23]. Specifically, an excitation wavelength of 780 nm and a power setting of 10% maximum excitation power were used. The collected emission wavelength range was 451–697 nm. Images were collected at a magnification of 20x and a *z*-stack (depth) increment of 5 *μ*m. In order to calculate the percentage of area covered by nanoshells, ImageJ imaging software was implemented after image acquisition. Recent research has shown that ImageJ can be used to analyze signal intensity of silica-gold nanoshells under different imaging systems [11, 24]. Each pixel in the images had an intensity value in the range of 0–255. To determine the nanoshell level in each image, an intensity threshold of 30 was used to separate areas that did not have nanoshells (≤30) from those that did have nanoshells (>30). The value of 30 was chosen because images of negative controls were found to have a maximum intensity of 30. The number of pixels that were above the threshold value was then used to calculate the area of each image that contained nanoshells. 

### 2.4. Darkfield Hyperspectral Imaging of Human Breast Tissue Slices

 To confirm the presence of nanoshells on the surface of the tissues, HER2-positive cancerous, HER2-negative cancerous and normal tissue samples were incubated with nanoshells as previously described. A thin layer of pathological ink was placed on the tissue surface for orientation. The tissues were embedded in OCT media (BBC chemical) and frozen rapidly over dry ice. The specimens were cut at a section thickness of 8 *μ*m using a Leica CM1850 UV cryostat. Cancerous specimens were sectioned at −20°C and normal specimens at −30°C. The different temperatures were used to maintain optimal tissue morphology as recommended by Leica. Additionally, Magalhães et al. reported on the use of different temperatures to slice normal and cancerous tissue [25]. The sections were immediately placed on superfrost slides (Fisher Scientific) and allowed to dry overnight. The next day the tissue slices were imaged with a 10x objective on an Olympus darkfield microscope equipped with a Cytoviva high-resolution illuminator. Hyperspectral images of the tissue slices were taken using a hyperspectral camera that provides both spatial and spectral data for each image.

 Spectral data of each field of view (FOV) was used to determine if nanoshells were present on each slice of tissue. Comparisons were made between tissue surfaces and tissue beyond the surfaces to determine the presence of nanoshells; spectral data from tissue that was not incubated with nanoshells was also used as a negative control. 

### 2.5. Macroscopic Imaging of Human Breast Tissue Specimens

Normal and HER2-positive cancerous breast tissue specimens (from patients who had and had not received neoadjuvant chemotherapy) were imaged using a Zeiss Discovery. V8 stereomicroscope equipped with a VisiLED MC1000 light source. For macroscopic imaging of breast tissue specimens, a thin plastic black stage was placed beneath a glass coverslip to enable ease of tissue placement and to provide a consistent black background among all samples. The specimens (controls and respective nanoshell-labeled counterparts) were placed alongside each other on top of the coverslip. Images were taken at both 1x and 2x magnification under the same lighting conditions. 

### 2.6. Reflectance Confocal Microscopy Imaging of Human Breast Tissue Specimens

Following widefield imaging, the aforementioned samples were prepared for microscopic analysis under reflectance confocal microscopy. For this component of the study, a Lucid VivaScope 2500 inverted confocal microscope was used. Samples were placed directly on glass slides that were modified by the addition of an adhesive 1 mm deep, 20 mm diameter silicon isolator (Invitrogen). To compress the tissue slightly and consistently among samples, an adhesive tissue cassette (Lucid, Inc.) was placed directly on top of the silicone isolators above the tissue specimens. Multiple images were taken at a power of 0.4 mW and at the same distance from the glass surface for both samples and controls. After reflectance imaging, the samples were prepared for histological processing. Additionally, reflectance intensity measurements were recorded using ImageJ processing software as formerly described [11].

### 2.7. Immunohistochemistry and Histology

Once images were collected under both stereomicroscopy and RCM imaging systems, normal and HER2-positive cancerous samples (with and without previous neoadjuvant chemotherapy) were embedded in OCT media and sectioned to a thickness of 5 *μ*m. Multiple sections from each specimen were prepared for either immunohistochemistry (IHC) or hematoxylin and eosin (H&E) staining. IHC for the HER2 antigen was executed using the Histostain Plus AEC Broad Spectrum Kit (Invitrogen) per manufacturer's instructions. H&E staining was also performed per manufacturer's instructions (Sigma Aldrich) for the alcoholic Eosin Y solution. For image acquisition, a standard brightfield microscope (Zeiss Axioskop 2 equipped with a Zeiss Axiocam MRc5 color camera) was used at a magnification of 20x.

## 3. Results

### 3.1. Distribution and Penetration of Gold Nanoshells in Intact Human Breast Tissue

**Figure 2:**
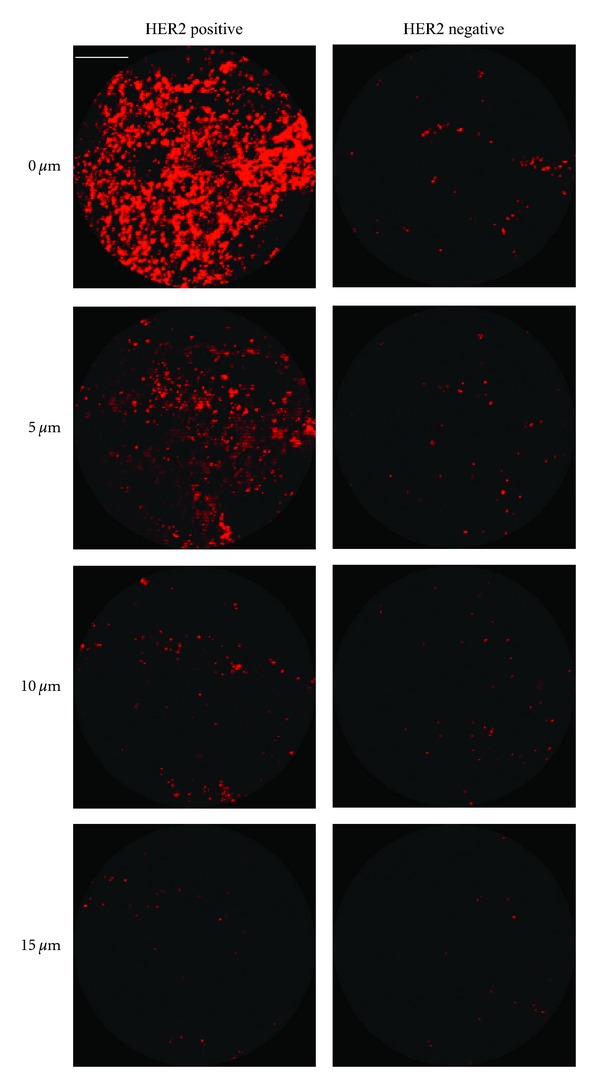
*Z*-stack two-photon luminescence images of HER2-positive and HER2-negative tissue incubated with HER2-targeted nanoshells for 5 minutes at 37°C. Each progressive image represents an increase in depth penetration of 5 *μ*m. Magnification = 20x. Scale bar = 50 *μ*m.

The goal of this study was to evaluate the distribution of anti-HER2-conjugated gold nanoshells on resected intact tissue specimens. For comparison, the nanoshell labeling between HER2-positive and HER2-negative tissue samples was evaluated using a two-photon imaging system. As previously reported, this imaging system is capable of enhancing and capturing the luminescence signature of the gold nanoshells [23] while also collecting a stack of images taken through the depth of the tissue of interest.  Figure 2 represents such images of HER2-positive and HER2-negative cancerous tissue samples incubated with HER2-targeted nanoshells. Each sequential increment in the *z*-direction represents 5 *μ*m into the tissue. Qualitatively, the first image (taken at the surface or at 0 *μ*m) in Figure 2 demonstrates that the nanoshells preferentially label HER2 receptors on the surface of the tissue. Additionally, Figure 2 displays decreased signal as the focal spot from the confocal microscope penetrates further into the tissue. This is believed to be due to a minimal number of nanoshells being able to penetrate the tissue in the limited amount of incubation time, thus decreasing signal collected beyond the surface. A quantitative difference of the nanoshell signal at the surface of the HER2-positive and HER2-negative tissue was calculated. Using ImageJ imaging software, it was determined that approximately 66% of the FOV for HER2-positive tissue was covered in nanoshells versus just 2% for the FOV of the HER2-negative tissue. This confirms the preferential labeling and visualization of HER2-positive tissue using anti-HER2 nanoshells.

**Figure 3:**
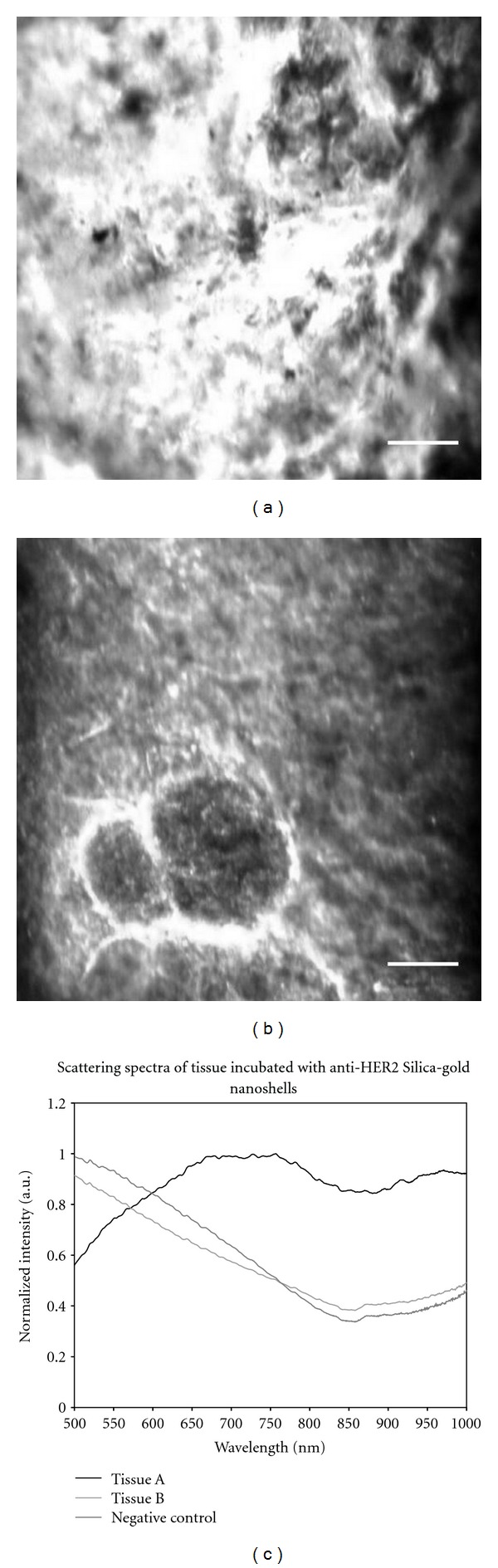
Darkfield images of HER2-positive tissue sectioned after incubation with anti-HER2-targeted silica-gold nanoshells. (a) Surface of HER2-positive tissue, (b) 24 *μ*m beyond the surface of the same tissue. (c) Scattering spectra of the fields of view depicted in (a) and (b). Additionally, spectra from the surface of HER2-positive tissue not incubated with silica-gold nanoshells are shown as a negative control. Scale bar = 50 *μ*m.

To further validate the surface binding of the nanoshells, hyperspectral images of different tissue sections were also acquired.  Figure 3(a) shows a representative surface of a HER2-positive tissue section after incubation with anti-HER2 nanoshells.  Figure 3(b) illustrates tissue 24 *μ*m beyond the surface of the same tissue. Spectra from multiple (*n* = 3) specimens that were incubated with anti-HER2 nanoshells were acquired, and analysis showed that tissues without nanoshells had very similar spectra across different patients.  Figure 3(c) displays the respective spectral information of each FOV shown in Figures 3(a) and 3(b). Additionally, the spectra of HER2-positive tissue without nanoshells have been included as a control. As can be seen in this graph, the spectra of the surface of the HER2-positive tissue incubated with anti-HER2 nanoshells are distinctive from that of the same tissue 24 *μ*m beyond the surface. In fact, the spectra of the tissue beyond the surface of the nanoshell-labeled specimen are very similar to the spectra of the surface of the control. These results support our findings that the targeted nanoshells primarily localized to the surface of the tissue.

### 3.2. Enhanced Optical Imaging of Intact **Ex Vivo ** Human Breast Cancer Tissue Using Gold Nanoshells

**Figure 4:**
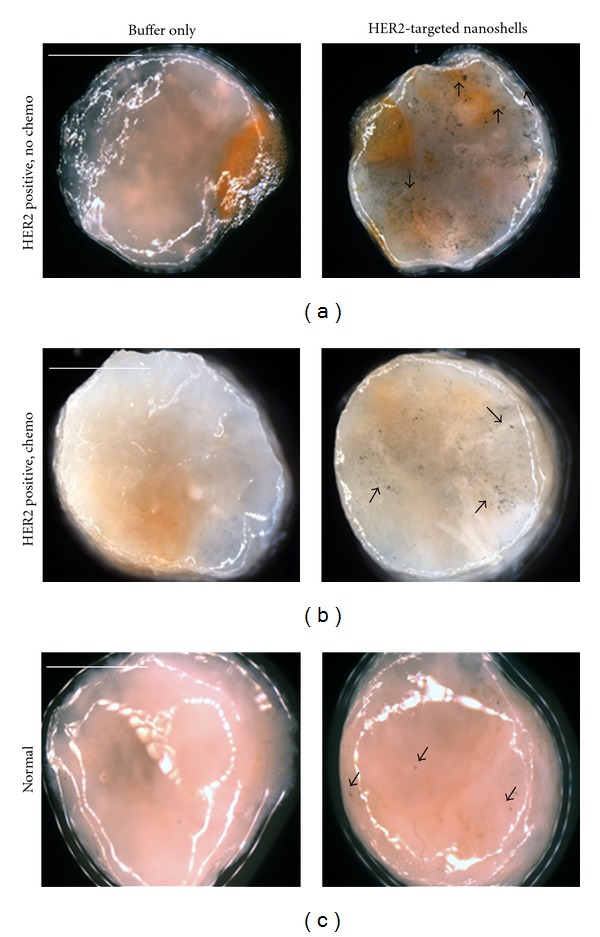
Raw stereomicroscope images of (a) and (b) HER2-overexpressing cancerous and (c) normal tissue incubated with either buffer or HER2-targeted nanoshells for 5 minutes at 37°C. Cancerous tissue taken from a patient (a) without chemotherapy and (b) following neoadjuvant chemotherapy. Arrows represent nanoshells. Images taken at 2x. Scale bars = 2.5 mm.

Based on previous results demonstrating the preferential labeling of HER2-targeted nanoshells on the surface of intact *ex vivo* HER2-positive tissue specimens, we assessed the potential of using a standard stereomicroscope to visualize this enhanced contrast. For this component of the study, human breast tissue specimens that overexpressed HER2 receptors at the time of patient diagnosis were evaluated and compared to normal breast tissue. Due to the ultimate goal of utilizing gold nanoshells to rapidly label tumor margins intraoperatively in diverse patient populations, we examined tissue from patients who had and had not undergone neoadjuvant chemotherapy. All tissue samples were incubated with either antibody diluent buffer or the anti-HER2-targeted nanoshells for 5 minutes at 37°C. As shown in Figure 4, which represents raw images taken with a stereomicroscope, intact tissue specimens incubated with antibody diluent alone showed no markings or features characteristic of nanoshells. However, tissue specimens incubated with the anti-HER2-targeted nanoshells demonstrate numerous particles on the surfaces of the tissues. Qualitatively, the HER2-positive tissue from the patient who did not undergo previous chemotherapy shows the greatest labeling with the targeted nanoshells. The HER2-positive tissue from the patient who did undergo neoadjuvant chemotherapy does demonstrate enriched nanoshell labeling when compared to normal tissue, though not to the same extent as the patient without previous chemotherapy. In contrast, the normal tissue shows the least amount of nanoshell labeling, and only a few areas of nanoshells can be visually perceived.

**Figure 5:**
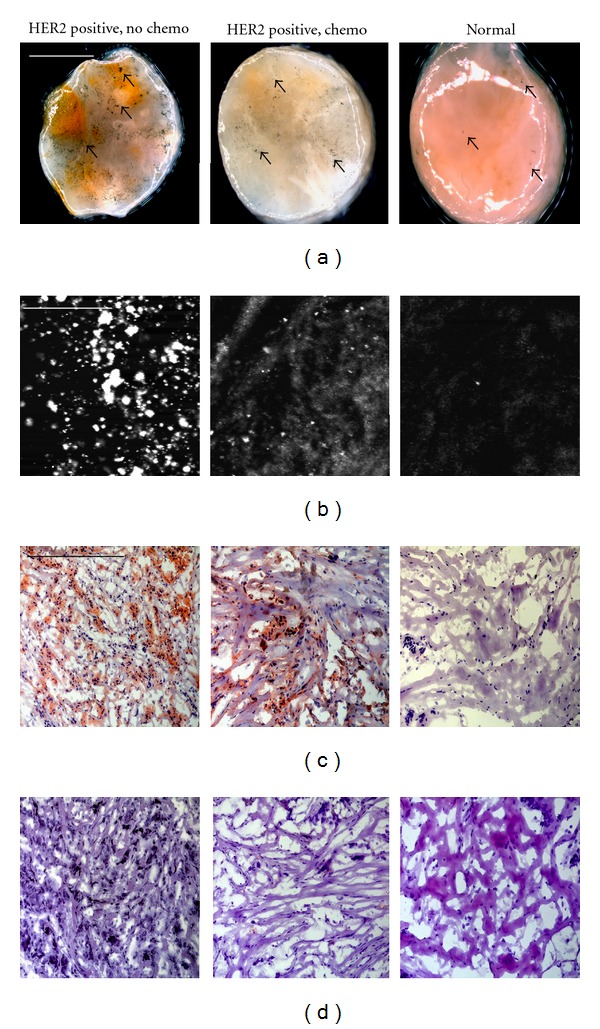
(a) Stereomicroscopic images of HER2-overexpressing breast tissue (with and without neoadjuvant chemotherapy) and normal breast tissue incubated with HER2-targeted nanoshells for 5 minutes at 37°C after contrast enhancement. Magnification at 2x; scale bar = 2.5 mm. Arrows represent nanoshells. (b) Respective reflectance confocal microscopy images of tissue samples from (a). Power = 0.4 mW and scale bar = 75 *μ*m. Respective (c) HER2 immunohistochemistry and (d) H&E results taken under brightfield microscopy under 20x magnification. Scale bar = 0.35 mm.

While the degree of nanoshell labeling can be visualized without image adjustments under a standard stereomicroscope, the superior extent of this labeling can be seen more clearly after a simple contrast enhancement using imaging software (ImageJ). As seen in Figure 5(a), the nanoshells are even more discernable against the tissue background regardless of inherent tissue constituents. 

To validate the enhanced nanoshell labeling seen by macroscopic imaging, the surfaces of the same tissue samples were also imaged using reflectance confocal microscopy (Figure 5(b)). Concurring with the stereomicroscopic images, we see dramatic nanoshell surface labeling when using targeted nanoshells with previously untreated HER2-positive tissue. For the HER2-positive sample that had formerly undergone chemotherapy, we also see enhanced nanoshell labeling, though to a lesser degree than the untreated sample as suggested by the stereomicroscopy results. The normal breast tissue displays the least amount of surface labeling with only minimal nanoshells evident with either imaging system. Reflectance intensity measurements (data not shown) were *∼*2.5 to 3 times greater for both the HER2-positive tissue sample receiving chemotherapy and for the HER2-positive tissue not receiving chemotherapy when compared to the normal tissue sample. 

Subsequent histological analysis shown in Figure 5(c) reveals that the distribution of HER2 receptors seen with nanoshell-enabled contrast corresponds to that seen with IHC against HER2. The HER2 expression seen by IHC is greater for the previously untreated HER2-positive tissue sample than for the sample that had undergone neoadjuvant chemotherapy. This is believed to be due to the effects of chemotherapy. Rasbridge et al. previously demonstrated that patient response to chemotherapy is highly variable, with patients previously negative for HER2 overexpression occasionally becoming positive after treatment and patients previously positive for HER2 overexpression subsequently becoming negative [26]. Although patient response to chemotherapy varies, tissues previously identified as overexpressing HER2 receptors during initial diagnosis, regardless of chemotherapy exposure, demonstrate enhanced nanoshell labeling over normal tissue. Additionally, H&E-stained sections of all tissue samples have been included (Figure 5(d)) to illustrate the microscopic characteristics and differences associated with cancerous versus noncancerous conditions.

## 4. Discussion

In this study we demonstrated the ability to use targeted gold nanoshells to rapidly improve visualization of a specific biomarker associated with disease aggression and progression (HER2) in intact *ex vivo* human breast tissue and confirmed binding location via confocal and darkfield hyperspectral microscopy. By utilizing silica-gold nanoshells designed as rapid diagnostic imaging agents, surgeons and pathologists may be able to realize tumor margin status directly in the operating room after both macroscopic and microscopic assessment. While multiple methods of intraoperative tumor margin detection are currently under investigation [27–31], we are developing an inexpensive and portable system for rapidly analyzing *ex vivo* specimens based on the desire to enhance current methodologies without delay in clinical translation due to regulatory concerns associated with *in vivo* systems.

The ability to enhance contrast of malignancy using topically applied agents has previously been demonstrated for oral and breast tissue using fluorescently labeled deoxy-glucose and epidermal growth factor (EGF) conjugates [32–34] as well as cervical tissue using fluorescently labeled gold nanoparticles targeted to EGF receptors [35]. However, these studies employed incubation times ranging from 20–45 minutes, which exceeds the length of time currently needed to obtain tumor margin status using frozen section histology. Additionally, the aforementioned studies utilized optical clearing agents, which may be necessary for particles that target intracellular biomarkers [36, 37]. Nevertheless, gold nanoshells targeted to extracellular biomarkers may offer more favorable opportunities for *ex vivo* intraoperative tumor margin detection without the need for lengthy incubation times or the use of optical clearing agents. 

Recently, we verified that silica-based gold nanoshells could be used to enhance contrast of both HER2-overexpressing cells and tissue sections within 5 minutes of incubation time [10, 11]. However, translating this technology towards clinical relevancy requires the ability to assess whole, unsectioned specimens. Here, we confirm that gold nanoshells, when targeted to HER2 receptors, can be used to distinguish intact HER2-overexpressing *ex vivo* tissue from normal tissue within the same incubation time, and we demonstrate that this difference can be observed macroscopically. These results are supported by microscopic imaging and immunohistochemistry against HER2.

By employing macroscopic imaging intraoperatively, clinicians may be better able to distinguish cancerous and normal breast tissue prior to further microscopic analysis and subsequent histological processing. Ultimately, this system could also be used for other diagnostic applications, for other anatomical locations, and for other biomarkers associated with disease. By facilitating fast and accurate tumor margin results intraoperatively as a supplement to current diagnostic methods, we expect to reduce the amount of time spent in surgery due to inadequate tissue removal. 

To translate these findings more readily to the clinic, we are presently developing a low-cost widefield imaging system that can be used to detect the overexpression of HER2 (and other extracellular biomarkers) through contrast enhancement provided by gold nanoshells. In addition, we plan to collect data from diverse patient populations and assess results with fresh tissue samples. In this way, the use of gold nanoshells may demonstrate widespread efficacy or be limited only to specific patient subsets.
